# Cardiac responses to exercise distinguish postural orthostatic tachycardia syndrome variants

**DOI:** 10.14814/phy2.13040

**Published:** 2016-11-24

**Authors:** Paolo T. Pianosi, Darrell R. Schroeder, Philip R. Fischer

**Affiliations:** ^1^ Department of Pediatric and Adolescent MedicineMayo ClinicRochesterMinnesota; ^2^ Biomedical Statistics and InformaticsMayo ClinicRochesterMinnesota

**Keywords:** Hypovolemia, orthostatic intolerance, stroke volume

## Abstract

We previously showed that one‐third of adolescents with postural orthostatic tachycardia syndrome (POTS) have hyperkinetic circulation. In a subsequent cohort, we compare participants with POTS grouped according to cardiac output ($\dot{Q}$) versus oxygen uptake ($\dot{V}O_{2}$) function, whose circulatory response to exercise lay at the lower end of this distribution. We hypothesized that such grouping determines the circulatory response to incremental‐protocol, upright, cycle ergometry by whatever blend of flow and resistance adjustments best maintains normal blood pressure. We reviewed data on 209 POTS participants aged 10–19 years (73% female) grouped as follows: $\dot{Q}-\dot{V}O_{2}$ < 3.20 L·min^−1^ per L·min^−1^ were designated low $\dot{Q}$ or hypokinetic variant (*N* = 31); normal‐$\dot{Q}$ had slopes between 3.21 and 7.97; hyperkinetic participants had $\dot{Q}-\dot{V}O_{2}$ slope >8 L·min^−1^ per L·min^−1^ (*N* = 32). Heart rate response to exercise was virtually identical in each group. Mean stroke volume (SV) rose normally in the hyperkinetic group (51 ± 38%); less in the normal $\dot{Q}$ group (22 ± 27%); but was flat in the low $\dot{Q}$ group (−7 ± 16%). Mean arterial pressure was similar at rest while systemic vascular conductance was flat from rest to exercise in the hypokinetic group, and by comparison rose more steeply in the normal $\dot{Q}$ (*P < *0.001) and in the hyperkinetic (*P = *0.02) groups. In conclusion, we identified a variant of POTS with a hypokinetic circulation maintained by a vasoconstricted state. We speculate that they cannot muster preload to augment exercise SV due to profound thoracic hypovolemia, and must resort to vasoconstriction in order to maintain perfusion pressure within working muscle.

## Introduction

Postural orthostatic tachycardia syndrome (POTS) is defined by symptoms of orthostatic intolerance associated with a physical sign, that is, excessive increase in heart rate (HR) on orthostatic challenge (Grubb 2008). It is a heterogeneous disorder of unknown etiology, more frequent in women, and most cases occur between the ages of 15 and 25 years (Benarroch 2008), implying onset often during adolescence (Johnson et al. 2010). A disease model of high, normal, and low‐flow POTS was proposed based on measurements of limb blood flow at rest (Stewart and Montgomery 2004). They reported that high‐flow POTS was characterized by inadequate peripheral vasoconstriction in both the supine and upright positions which exaggerated cardiac output as in other high‐output states. Our initial hypothesis was that if there were high limb blood flow, there ought to be high cardiac output, and whether this condition would persist during exercise. We demonstrated such a phenomenon (Pianosi et al. 2014) and labeled it hyperkinetic circulation. We next asked whether another variant of POTS existed among adolescents, analogous to the low‐flow variant described by Stewart and Montgomery, characterized by defects in *local* blood flow regulation in dependent limbs. They further postulated that mild absolute hypovolemia was also present and contributed to orthostatic intolerance (Stewart and Montgomery 2004). Thus, we hypothesized that there exists a subset of patients with POTS who exhibit a relatively low cardiac output ($\dot{Q}$) during exercise – low‐flow POTS. Those who demonstrate low‐output or hypokinetic responses to exercise must therefore regulate systemic arterial pressure primarily by vasoconstriction in order to maintain adequate muscle perfusion during exercise as a compensatory mechanism. Such circulatory adaptations imply systemic vascular conductance that would be *de facto* lower than participants with a relatively normal circulatory response to exercise, and diametrically opposed to high conductance found in hyperkinetic POTS.

## Methods

We retrospectively audited medical records of adolescents (12–19 years of age) seen in the Mayo Clinic Pediatric Diagnostic Referral Clinic from June 2012 to December 2014 with symptoms of chronic (≥6 months) fatigue, dizziness or orthostatic intolerance, who underwent both autonomic reflex and maximal exercise tests. We excluded those with alternative (other than POTS) medical diagnoses explaining their symptoms. There was no overlap among patients in this report and those from our prior studies. Testing was conducted for clinical indications. Mayo Clinic Institutional Review Board approved the study.

Patients were advised to discontinue vasoactive medications at least 1 day prior to tests, but in practice tests were typically done after longer (e.g., 48–72 h) washout, enough to allow clearance of midodrine (half‐life 3–4 h), (metoprolol [half‐life 3–4 h] or atenolol [half‐life 6–7 h]. Head‐up tilt table testing was performed by standard of care methods at Mayo Clinic (Thieben et al. 2007). Orthostatic tolerance was assessed by a 10‐min head‐up tilt table test and patients were diagnosed with POTS (Singer et al. 2012) if their ∆HR exceeded 39 beats·min^−1^. Part of their standard workup included routine urinalysis on a morning specimen. Records were reviewed and urine osmolality recorded. A minority of participants overall underwent echocardiography (only two in hypokinetic group), so these data could not be explored further. Participants performed a maximal exercise test on a cycle ergometer according to the Godfrey protocol of 1‐min incremental workloads (Godfrey 1974). We altered our testing protocol by choosing smaller work increments each minute (e.g., 10 vs. 15 watts·min^−1^) in order to obtain more measurements of $\dot{Q}$ during exercise. Ventilation and gas exchange were measured breath‐by‐breath at rest and throughout exercise using MedGraphics CPX/D (Breeze software; Medical Graphics Corp, St. Paul, MN) which employs a Pitot tube to measure flow, electronically integrated to give minute volume ($\dot{V}E$), yielding breath‐breath $\dot{V}O_{2}.$ Exhaled gases were measured by mass spectrometry (Perkin‐Elmer, Pomona, CA). Cardiac output was measured using a closed‐circuit, acetylene rebreathing (Triebwasser et al. 1977). $\dot{V}O_{2}$ and $\dot{Q}$ were measured at rest with the subject seated on the cycle ergometer; $\dot{Q}$ was also measured during the initial exercise workload then during alternate workloads until HR reached 160–170 min^−1^ or respiratory quotient exceeded 1.1. This was done at the discretion of the supervising physician in order to obtain at least three measures of $\dot{Q}$, without causing the patient undue dyspnea from rebreathing. HR was monitored with a 12‐lead continuous ECG (Quinton Cardiology Bothell, WA). Blood pressure was measured by auscultation prior to each $\dot{Q}$ determination. Oxygen saturation was continuously monitored by pulse oximetry (Nellcor OxiMax N‐600X, Medtronic, Minneapolis, MN). Patients were strongly encouraged to exercise to volitional exhaustion such that highest $\dot{V}O_{2}$ sustained over 15 sec was taken as peak $\dot{V}O_{2}.$ Peak $\dot{V}O_{2}$ was not an aim of this report, and analyses were conducted on submaximal data. Ventilatory anaerobic threshold was computed by the V‐slope method (Beaver et al. 1986). Stroke volume (SV) was computed as $\dot{Q}$/HR to assess inotropic response while HR was regressed versus $\dot{V}O_{2}$ to measure chronotropic response to exercise.

**Figure 1:**
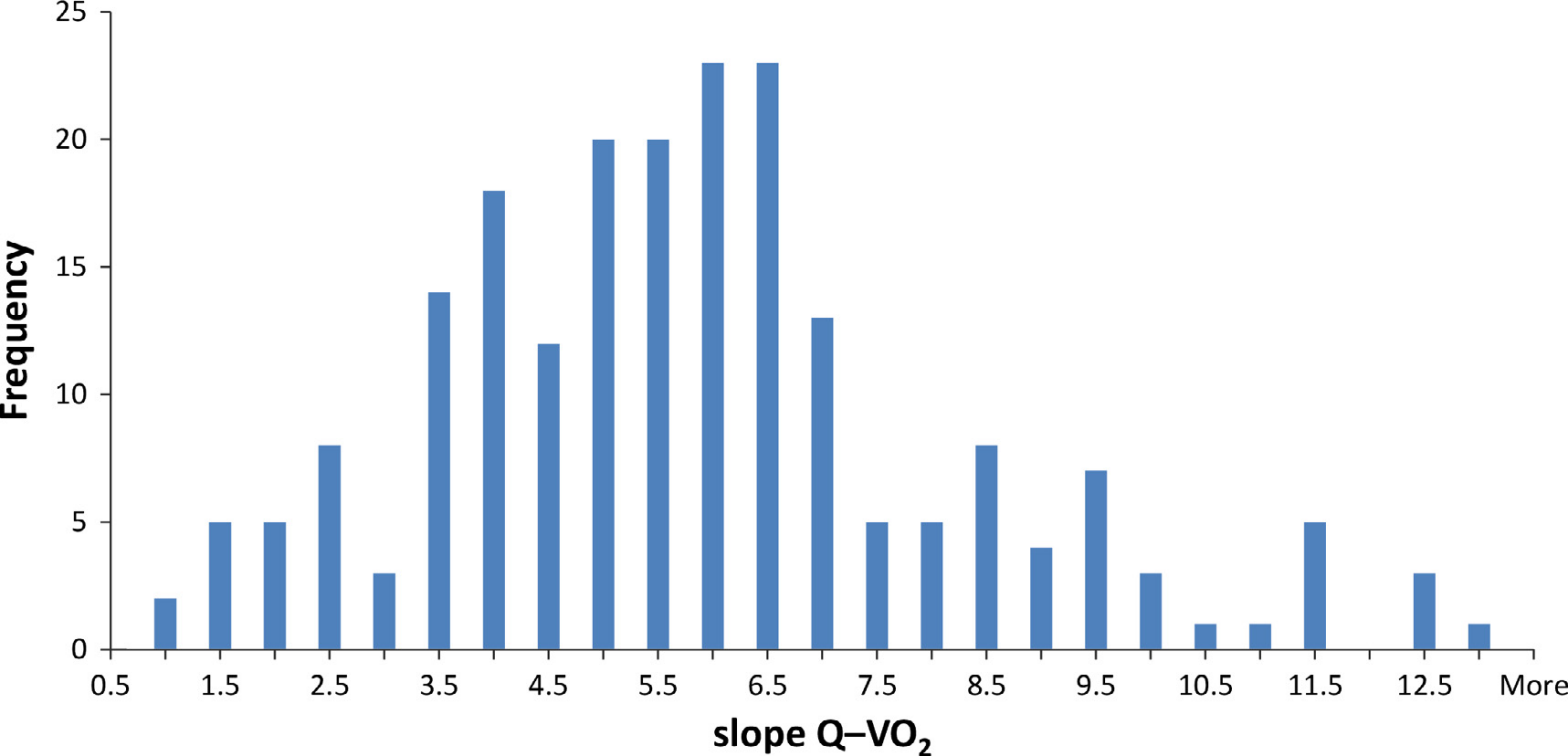
Frequency distribution of slopes for cardiac output versus oxygen uptake in patients with POTS. This report compares participants comprising left tail of graph, participants comprising large central hump of histogram, and those comprising the long rightward tail.

**Figure 2:**
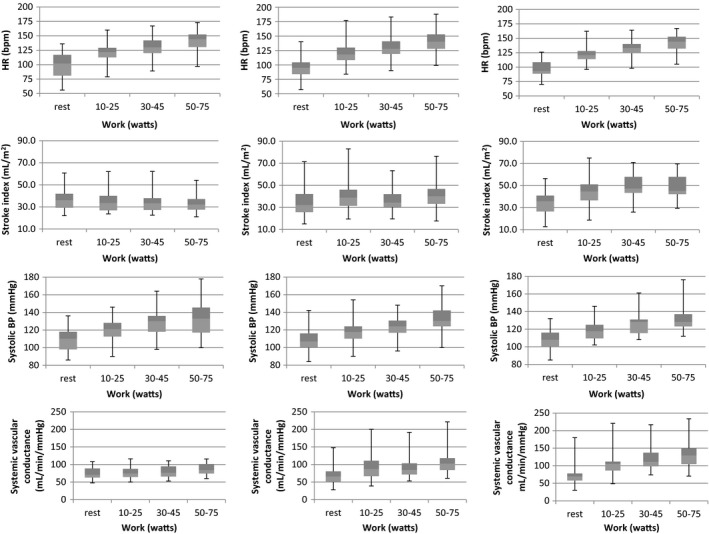
Box and whisker plots (left to right, top to bottom) of HR (A–C), stroke index to minimize effect of body size (D–F), mean arterial pressure (G–I), and systemic vascular conductance (J–L), measured at rest, and during progressive exercise grouped separately for POTS participants as defined in methods, with low‐flow variant in left column, normal $\dot{Q}$ in center, and hyperkinetic group in right column.

Values are reported as mean ± SD. Linear regression was used to compute $\dot{Q}$ versus $\dot{V}O_{2}$. The preponderance of females precluded separate analyses by sex. After data analysis for our report on hyperkinetic circulation in POTS, it became apparent that identifying a group of patients with blunted cardiac output would be more challenging – chiefly where to set the cut‐off point separating those with “normal” cardiac output response to exercise from those with blunted $\dot{Q}-\dot{V}O_{2}$ relationship during exercise. We set this threshold at $\dot{Q}-\dot{V}O_{2}$ ≤ 3.20 L·min^−1^ per L·min^−1^ for the following reasons: (1) visual inspection of Figure 1; (2) $\dot{Q}-\dot{V}O_{2}$ in healthy adolescents is ~5.5 L·min^−1^ per L·min^−1^ and in our sample as a whole was 5.59 ± 2.38 L·min^−1^ per L·min^−1^. Thus, one SD below this was 3.21 and one SD above this was 7.97; (3) our prior definition of hyperkinetic circulation was >7.5 L·min^−1^ per L·min^−1^. Data points to compute slope of the $\dot{Q}$ versus $\dot{V}O_{2}$ relationship from rest to exercise were obtained in participants as follows: three measurements of $\dot{V}O_{2}$ and $\dot{Q}$ in 52% of participants; four measurements in 42%; five in the remainder.

All analyses were performed using SAS software version 9.3 (SAS Institute Inc., Cary, NC). Participant characteristics were compared between groups using Student's *t*‐test. The cardiovascular responses to exercise were analyzed using repeated measures analysis ANOVA (PROC MIXED). For these models, a first‐order, autoregressive covariance structure was used to account for the repeated measures.

## Results

There were 209 participants in total, none were anemic and physical characteristics of the three groups were very similar (Table 1). Urine osmolality was similar among groups: 609 ± 215, 565 ± 227, and 648 ± 236 mOsm·kg^−1^ (ANOVA *p = *0.2) in the hypokinetic, normal $\dot{Q}$, and hyperkinetic groups, respectively. Figure 1 shows the frequency distribution for $\dot{Q}$ versus $\dot{V}O_{2}$ slopes for all patients with POTS. The cluster at the left end of the distribution (lower values) from our sample comprised ~15%, designated the hypokinetic group. Resting $\dot{Q}$ was similar in all three groups (Table 1, *P* = 0.19). Linear fits for $\dot{Q}$ versus $\dot{V}O_{2}$ were acceptable with a median *r* ^*2*^ value of 0.95 (IQR, 0.89–0.99) for the $\dot{Q}-\dot{V}O_{2}$ relationship. On average, $\dot{Q}$ rose 5.35 ± 1.12 L·min^−1^ in the normal $\dot{Q}$ group, 2.25 ± 0.74 L·min^−1^ in the hypokinetic, and 9.74 ± 1.39 L·min^−1^ in the hyperkinetic subgroup, for each L·min^−1^ $\dot{V}O_{2}$ during progressive exercise.

**Table 1:** Physical characteristics at rest, according to POTS variant

Characteristic	Low flow	Normal $\dot{Q}$	Hyperkinetic	Low flow	Normal $\dot{Q}$	Hyperkinetic
	Males			Females		
M:F	7	42	10	24	103	23
Age, years	14.6	15.0	15.5	15.4	15.5	15.3
Height, cm	178	173	174	167	166	164
Weight, kg	57	66	67	60	58	56
BSA, m^2^	1.71	1.78	1.79	1.66	1.64	1.61
BMI, kg·m^−2^	18.1	21.6	22.1	21	20.9	20.8
Hgb, g·dL^−1^	15.4	14.8	14.8	13.5	13.4	13.4
Systolic BP (mmHg)	108	110	114	110	107	106
Diastolic BP (mmHg)	74	72	70	71	71	70
$\dot{Q}$ seated (L·min^−1^)	6.63	6.39	6.75	6.20	5.26	5.35
$\dot{V}O_{2}$ seated (mL·min^−1^)	367	371	372	311	291	318
Supine HR (bpm)	77	75	73	79	75	76
∆HR on HUT (bpm)	54	52	50	50	50	52

Data presented as mean ± SD. HUT, head‐up tilt table test result.

Exercise data are shown in Table 2. Cardiovascular responses to exercise in each group are shown in Figures 2. By definition, $\dot{Q}$ trajectory with increasing work (i.e., $\dot{V}O_{2}$) differed between groups (group‐by‐work interaction *P *= 0.001). HR rose (Fig. 2A–C) significantly with increasing work for all groups (*P *< 0.001) with no evidence suggesting that the increase in HR differed between groups (group‐by‐work interaction *P *= 0.87). Similarly,∆HR/∆$\dot{V}O_{2}$ did not differ significantly (*P = *0.38) across the three groups (Table 2). The effect of increasing work on SV differed significantly between groups (group‐by‐work interaction *P *< 0.001). There was no significant SV recruitment with exercise (*P *= 0.80) in the low‐flow group, an early but blunted SV increase in the normal $\dot{Q}$ group, and a brisk initial rise in SV followed by a plateau in the hyperkinetic group (Fig. 2D–F). Tracking changes in SV from rest to exercise was complicated by fact that SV of many participants, particularly those in low‐flow group, often fell with increasing exercise intensity, even if there was an initial rise during transition from rest to light exercise. Therefore, ∆SV was computed as the difference between the last measurement minus resting value, results of which are shown in Table 3. MAP was similar across the board at baseline (*P = *0.68)) and rose with increasing work (*P *< 0.001) in all groups but with no significant group‐by‐work interaction effect (*P *= 0.65) (data not shown). The same held true for systolic blood pressure; it rose systematically with increasing work in all groups ((*P *< 0.0001), but with no differences between groups (*P *= 0.31) or work‐by‐group interaction (*P *= 0.5) (Fig. 2G‐I). On the other hand, change from rest in systemic vascular conductance (Fig. 2J–L) with increasing work differed significantly across groups (group‐by‐work interaction *P *< 0.001). There was no significant effect of workload in the low $\dot{Q}$ variant (*P = *0.18), whereas conductance rose in the normal $\dot{Q}$ group (*P < *0.001) and hyperkinetic groups (*P *= 0.02).

**Table 2:** Exercise data, according to POTS variant

	Low flow	Normal $\dot{Q}$	Hyperkinetic
$\dot{V}O_{2}$ (L·min^−1^)	1.77 ± 0.50	1.84 ± 0.55	1.78 ± 0.46
HR (bpm)	186 ± 17	190 ± 11	191 ± 7
Systolic BP (mmHg)	149 ± 22	152.3 ± 17	155 ± 15
Diastolic BP (mmHg)	74 ± 13	71 ± 11	74 ± 12
${\dot{V}}_{E}$ (L·min^−1^)	71 ± 18	75 ± 19	73 ± 22
O_2_ pulse (mL·beat^−1^)	9.58 ± 2.80	9.67 ± 2.85	9.27 ± 2.39
Work (watts)	125 ± 38	136 ± 44	126 ± 44
VAT (% peak $\dot{V}O_{2}$)	57 ± 10	57 ± 12	58 ± 8
∆HR/∆$\dot{V}O_{2}$ (beat·L^−1^)	64 ± 16	69 ± 23	72 ± 24

Data are means ± SD. All values at peak exercise, except VAT (ventilatory anaerobic threshold) and ∆HR/∆$\dot{V}O_{2}.$ There were no significant differences.

**Table 3:** HR and SV data, specifically change from rest to highest exercise values, according to POTS variant

	Low flow	Normal $\dot{Q}$	Hyperkinetic
∆SV (mL)	−7 ± 13	10 ± 13	25 ± 13
∆SV (%)	−7 ± 16	22 ± 27	51 ± 38
HR (bpm)	142 ± 18	148 ± 16	146 ± 12

Data are means ± SD. No difference in HR (*P *> 0.10) but highly significant differences (*P *< 0.0001) differences in both absolute and relative (%∆) from rest changes in SV.

## Discussion

On average, $\dot{Q}$ rises ~5–6 L·min^−1^ per L·min^−1^ increase in $\dot{V}O_{2}$ during submaximal exercise in adults (Astrand Po 1970; Beck et al. 2006) and children (Godfrey 1974; McNarry et al. 2011). We demonstrated that the slope of $\dot{Q}$ versus $\dot{V}O_{2}$ was low in ~15% of our patients with POTS, relatively normal in approximately two‐third of patients, and abnormally high in the remainder of this convenience sample of participants. These findings reflect consensus that POTS is a heterogeneous disorder while at the same time underscore and elucidate different compensatory mechanisms for specific circulatory perturbations. POTS has been categorized in a few different ways, for example, neuropathic, neurohumoral, or circulatory variants (Medow and Stewart 2007; Raj 2013) and our data extend the model proposed by Stewart and Montgomery (Stewart and Montgomery 2004) to exercise. The significance of our findings is that exercise has become a cornerstone of treatment (Kizilbash et al. 2014) regardless of the nature of the underlying pathophysiology. Our results support a phenotypic distinction among adolescents with POTS based on circulation during exercise: one that maintains the normal coupling between $\dot{Q}$ and $\dot{V}O_{2}$, another in whom $\dot{Q}$ rises more than expected, and now a third in whom $\dot{Q}$ rises *less* than expected, with respect to $\dot{V}O_{2}.$ How $\dot{Q}$ is matched to $\dot{V}O_{2}$, can provide insight into pathophysiology of each variant. The distribution of $\dot{Q}$ versus $\dot{V}O_{2}$ among patients in this study reflects how we defined the three variants from a presumed Gaussian distribution of $\dot{Q}$ versus $\dot{V}O_{2}$, that is, mean ± 1 SD. However, if one pools these data with those from our earlier study, the distribution of low, normal, and high $\dot{Q}$ for $\dot{V}O_{2}$ among adolescents with POTS more closely approximates 66% normal $\dot{Q},$ ~10% low flow, and a skewed, rightward tail comprised of ~25% hyperkinetic, depending on choice of cut‐points.

There were no differences at rest among participants in each group, they experienced similar changes in blood pressure or HR in response to head‐up tilt table tests; nor did. Participants were in a similar state of (de)hydration, judging from urine osmolality as an indicator of circulating volume status. Fitness level (whether judged by ventilatory anaerobic threshold or peak $\dot{V}O_{2}$) differ among the three groups. This prompts the questions how could $\dot{V}O_{2}$ rise to similar levels despite such differences in $\dot{Q}$? The answer can be deduced by computing arteriovenous O_2_ content changes from rest to the highest exercise level at which of $\dot{Q}$ was measured. There were clear work‐by‐group differences and interaction in this parameter (*P < *0.0001), with significant increase in the hypokinetic and normal $\dot{Q}$ participants (*P* < 0.001 for both), but no change in the hyperkinetic group (*P = *0.45). The latter was not unexpected given earlier findings (Holmgren et al. 1957; Pianosi et al. 2014). Once exercise began, participants’ cardiovascular responses clearly began to diverge (Fig. 2G–I) and plots of systemic vascular conductance during exercise resulted in clear separation between groups. An intact sympathetic nervous system has a variety of countermeasures to respond to alterations in systemic arterial pressure (Rowell and O'Leary 1990). In the case of hyperkinetic POTS, where there is a failure of appropriate vasoconstriction in “unnecessary” vascular beds during exercise, cardiac output is augmented to maintain perfusion pressure. In contrast, hypokinetic POTS must regulate systemic arterial pressure primarily by vasoconstriction in order to maintain adequate muscle perfusion during exercise, that is, systemic vascular conductance must be low during exercise, confirming our hypothesis. Our results imply that blood pressure is the prime variable regulated.

We reasoned that a model of POTS based on resting peripheral blood flow into “low‐flow”, “normal‐flow”, and “high‐flow” (Stewart and Montgomery 2004), ought to have a corollary on exercise. The blunted $\dot{Q}$ response to exercise was achieved by relative tachycardia with virtually no SV recruitment in these participants. SV typically rises at onset of exercise, reaches a plateau in moderate exercise, but may fall during heavy exercise in adults (Higginbotham et al. 1986; Gonzalez‐Alonso 2008). There is a dearth of such data in pediatric participants though recent work indicates SV may progressively increase right up to peak exercise in healthy girls involved in regular and intense, physical activity (McNarry et al. 2011). It may fall in heavy exercise in unfit girls (in particular) because of differences in cardiac filling and Frank–Starling relationship compared with males (Fu et al. 2004). POTS results in recognizable circulatory changes: reduced circulating blood or plasma volume (Raj et al. 2005), smaller heart resulting in low SV, and relatively rapid HR (Fu et al. 2010). The Frank–Starling mechanism plays a key role in SV recruitment during light to moderate exercise (Plotnick et al. 1986). While many of the above studies were done in young adults with POTS, Rowland et al. (2009) found no differences in inotropic or lusitropic responses to incremental exercise despite higher SVI in athletic versus non‐athletic boys, and concluded that greater aerobic fitness in trained participants reflected volume expansion of the circulation rather than better ventricular function. Thus, generous circulating blood volume is a prerequisite for healthy SV response, regardless of age. Patients with POTS have ~13% deficit in plasma volume, ~34% RBC volume, and ~17% total blood volume compared to healthy controls (Raj and Robertson 2007). Therefore, one can reasonably presume that patients with POTS, particularly if deconditioned (the majority), have varying degrees of low intravascular volume status. Hypokinetic circulation during exercise may be the exercise‐equivalent of the low‐flow group at rest described by Stewart et al. who share phenotypic features with our participants. Mechanistic studies of limb blood flow at rest in this variant showed a tendency to lower calf blood volume, increased vascular resistance, and reduced venous capacitance, leading the authors to describe such individuals as existing in a chronically vasoconstricted state (Stewart et al. 2006). Our novel observations during exercise are entirely consistent: central hypovolemia compounded by an inefficient muscle pump (Stewart et al. 2004) reduced preload at the start of exercise, preventing SV recruitment.

There are limited options to maintain perfusion pressure in exercising muscle under such circumstances other than greater systemic vasoconstriction. Conversely, the healthy SV response in the hyperkinetic group implies these participants do not experience thoracic hypovolemia; nor are they de facto deconditioned. Their higher conductance implies hyperkinetic $\dot{Q}$ participants are unable to appropriately vasoconstrict and direct blood flow to where it is most needed – famine in the midst of abundance. Muscle pump preserved preload for these participants despite lower body pooling of blood (Stewart et al. 2006). In contradistinction, the hypokinetic variant must have experienced profound thoracic hypovolemia (Stewart and Montgomery 2004), similar urine osmolality notwithstanding, and struggled to maintain perfusion pressure to working muscle in the face of inability to boost $\dot{Q}$ sufficiently – starvation in the true sense. It is clear that differences in regional hemodynamics observed during head‐up tilt (Stewart et al. 2003) also exist during exercise. Low $\dot{Q}$ for $\dot{V}O_{2}$ implies increased O_2_ extraction at the level of exercising muscle, and arteriovenous O_2_ content differences derived from the Fick equation were systematically higher in hypokinetic POTS participants (data not shown). There was no difference in O_2_ pulse ($\dot{V}O_{2}$/HR) at peak exercise. Thus, hypokinetic $\dot{Q}$ participants must have had significantly greater O_2_ extraction at peak exercise in order to achieve similar peak $\dot{V}O_{2}$, assuming they did not recruit additional SV during heavy exercise, unlikely given their SV trajectory in Figure 2D. Such a response is diametrically opposed to the reduced extraction seen in hyperkinetic participants (Pianosi et al. 2014). Abnormalities of local blood flow regulation likely contribute to this contrast (Stewart and Weldon 2001) as part of global autonomic dysregulation. It seems that functional sympatholysis does not occur in this POTS variant.

Metabolite‐sensitive afferent neurons within the skeletal muscle are stimulated during exercise and evoke a reflex increase in sympathetic nerve activity to the heart and vasculature, known as the muscle metaboreflex (Murphy et al. 2011). It normally elicits an increase in blood pressure mainly via a marked increase in $\dot{Q}$ due to a combination of increases in both HR and SV during submaximal dynamic exercise (Kim et al. 2005b). Regulation of BP in the setting when O_2_ delivery to working muscle competes with maintaining MAP becomes a complex balancing act between arterial baroreflex and muscle metaboreflex (Ichinose et al. 2014). The arterial baroreflex normally buffers or modulates the muscle metaboreflex (Benarroch 2008), but low‐flow POTS participants behave as if they were barodenervated in as much as they maintain higher mean arterial pressure at the same time they display relative tachycardia, as do patients with heart failure (Kim et al. 2005a) (who also have a blunted $\dot{Q}-\dot{V}O_{2}$ relationship during exercise). Alternatively, the arterial baroreflex may act *in concert with* the muscle metaboreflex during upright exercise in a hypovolemic state to (a) reduce vasodilatory response to rising BP; (b) increase muscle sympathetic nerve activity; and (c) increase gain of muscle sympathetic nerve activity (Rowell and O'Leary 1990). Either pathway suggests aberrant central integration of competing afferent information.

The chief limitation of our study is that, we have no measure of blood or plasma volume. Urine osmolality was assessed in the vast majority of participants but time of day (and therefore fluid intake) was not standardized. Moreover, we are not aware of any studies documenting volume status in adolescents with POTS. Many reports from Stewart et. al. included adolescents, but most that involved invasive procedures, for example, catheterization for measures of cardiovascular performance, were done of necessity only in adults – HR criteria to define POTS being a perfect example. We acknowledge cardiovascular physiology will have certain unique features in pediatric subjects and caution extrapolating adult data even to adolescents is warranted. Nevertheless, data obtained in adults with POTS is internally consistent with our observations, which substantiate a circulatory phenotypic differentiation of POTS which was deduced in patients aged 14–21 (median 17.2) years (Stewart and Montgomery 2004). Thus, we can only speculate that participants suffered from varying degrees of intravascular volume contraction. We considered SV response to exercise as an indicator of inotropic capability, but recognize that such an inference presumes a similar change in end‐diastolic volume and systolic blood pressure in all groups. We have argued that preload (end‐diastolic volume) was low in the hypokinetic group, implying that systolic function may actually be preserved in these patients. We can only verify similar systolic BP but cannot verify similar contractility since we had echocardiographic data on few participants, but the underlying presumption is that the only deficit in this regard was a small heart in many participants (Fu et al. 2010). The normal $\dot{Q}-\dot{V}O_{2}$ relationship during exercise is well established (Astrand Po 1970) although it may become curvilinear and asymptotic near maximal exercise (Beck et al. 2006). Our $\dot{Q}$ measurements were restricted to light or moderate exercise, which may have resulted in overestimation of the change in $\dot{Q}$ for $\dot{V}O_{2}$ by limiting observations to subthreshold exercise.

In conclusion, we have now extended the “low‐flow”, “normal‐flow”, and “high‐flow” classification of POTS based on supine calf blood flow measurements to upright exercise. We propose this may be a useful paradigm particularly since exercise in an essential part of any rehabilitation program for POTS. Our observations have obvious therapeutic implications too. Although increased fluid intake, high‐salt diet, and exercise are intuitive standards of treatment, classification by cardiovascular response to exercise would allow one to tailor nonpharmacologic, and perhaps pharmacologic, management.

## Conflict of Interest

There are no conflicts of interest, financial or otherwise.
